# Extensor Tendons Rupture after Volar Plating of Distal Radius Fracture Related to a Dorsal Radial Metaphyseal Bone Spur

**DOI:** 10.1155/2018/8351205

**Published:** 2018-02-28

**Authors:** M. O. Abrego, F. L. De Cicco, J. G. Boretto, G. L. Gallucci, P. De Carli

**Affiliations:** Trauma and Orthopedics Institute “Carlos E. Ottolenghi”, Italian Hospital of Buenos Aires, Buenos Aires, Argentina

## Abstract

Extensor tendon ruptures due to volar plating in distal radius fractures have mostly been described in relation with technique failures such as screw prominence and drill penetration. We report the case of a 71-year-old female with a C2 distal radius fracture with severe dorsal metaphyseal comminution. The patient underwent surgical treatment with reduction of the large fragments and fixation with a volar locking plate; the small dorsal metaphyseal nonarticular fragments were not reduced. Six months later, the patient developed extensor digitorum communis (EDC) rupture and extensor indicis proprius (EIP) laceration in coincidence with the dorsal comminution turned into a bony spur. The possible association between the extensor tendon injury and the dorsal residual metaphyseal bony spur in the distal radius fractures is unusual but should be taken into account in fracture patterns presenting dorsal comminution.

## 1. Introduction

Volar plating is established as the gold standard treatment for distal radius fractures [1]. Even fractures involving dorsally displaced fragments can be treated with volar plates, decreasing the high rates of extensor tendons injuries due to dorsal plating [2]. Nevertheless, extensor tendinous complications following volar plating have been reported. Reported complications are irritation, adhesion, tenosynovitis, laceration, and even tendon rupture [3]. They are mostly associated with screw prominence and drill penetration of the dorsal cortex during screw fixation [4]. Extensor tendons rupture due to dorsal metaphyseal bony spur as a consequence of dorsal comminution is unlikely to happen. We present a case of rupture and laceration of extensor digitorum communis and extensor indicis proprius, respectively, as a complication of a distal radius fracture treated with a volar locking plate and in coincidence with dorsal metaphyseal exostoses secondary to dorsal metaphyseal comminution.

## 2. Case Report

**Figure 1:**
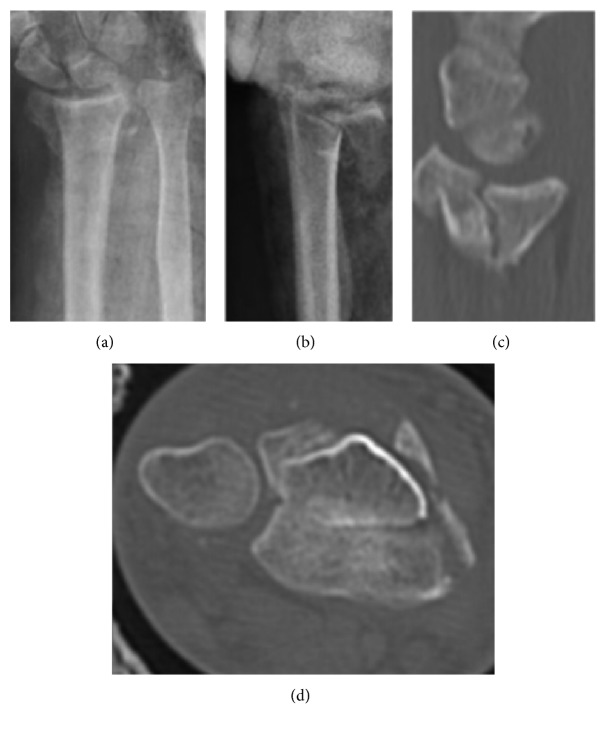
Preoperative X-rays, anteroposterior and lateral view of the 23C2 fracture (a, b). CT scan showing articular damage (c, d).

**Figure 2:**
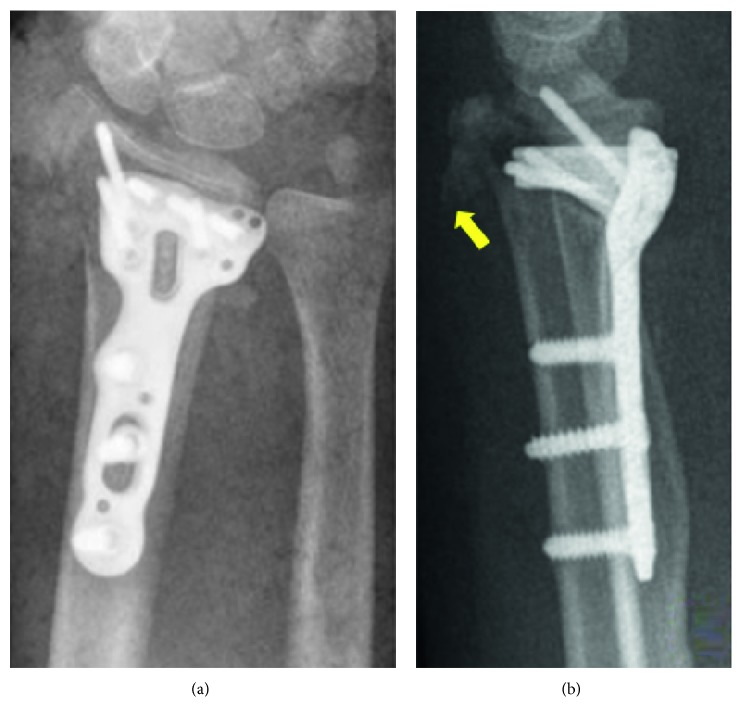
Immediate postoperative X-rays showing internal fixation (a). Lateral view showing dorsal metaphyseal comminution (arrow) (b).

A 71-year-old right hand dominant female patient with no comorbidities was admitted in our institution after suffering a fall from her own height. Plain radiographs and CT scan were performed. The patient had a C2 fracture according to AO/ASIF classification with dorsal metaphyseal comminution (Figure 1). Seven days later, the patient underwent surgical treatment: using a volar approach, open reduction and internal fixation with volar locking plate was performed. Drilling was performed only through the volar cortex without violating dorsal cortex as we usually do when performing this technique, in order to avoid extensor tendon erosion. Attention was specially paid to restoration of the articular surface and radial bone angles. Dorsal fragments from metaphyseal comminution were left in site, maintained by the dorsal periosteum. Postoperative X-rays showed adequate fracture reduction. However, dorsal metaphyseal fragments were larger than usual and displaced in a perpendicular plane in relation with the dorsal radial cortex (Figure 2). The patient was splinted for 15 days and then moved onto a removable wristband, starting rehabilitation protocol. Three-month follow-up showed that the patient's wrist had full-range motion, no pain, and the Disabilities of the Arm, Shoulder and Hand (DASH) score of 37.

Six months after surgery, the patient's DASH score improved to 9, maintaining a full range of motion, but she addressed dorsal wrist tenderness, with incomplete extension of the index finger. Her Visual Analogue Scale (VAS) for pain at rest was 1 but increased to 4 with activity. Normal “tenodesis effect” was reported, but the patient could not further extend her index finger with the hand on top of a table, as she could do with the other three ulnar fingers.

**Figure 3:**
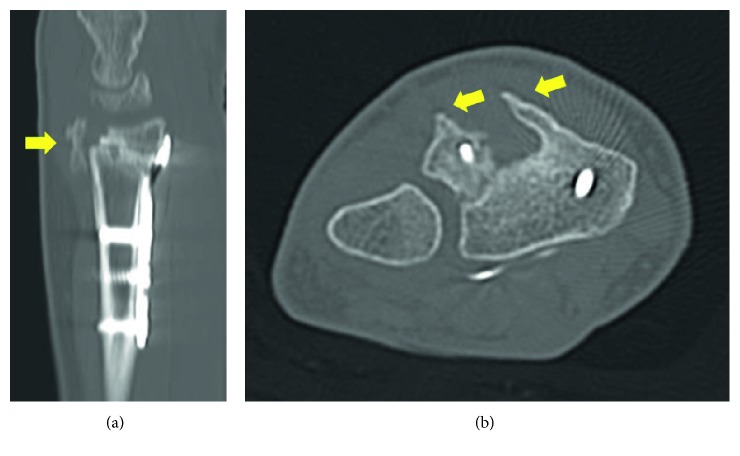
CT scan performed at 6-month follow-up. The arrows show dorsal bone spur.

**Figure 4:**
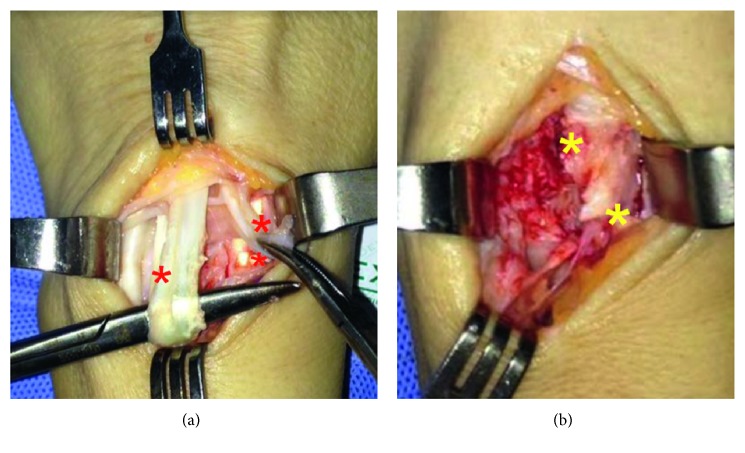
EDC laceration and EIP rupture (a). Dorsal exostoses (b).

Musculoskeletal ultrasound showed the presence of tenosynovitis of the fourth compartment and thinning of the index extensors at the radioulnar space, compatible with tendon rupture. New X-rays and CT scan showed sequel bone spicules at the dorsal epiphysis of the radius (Figure 3). Fracture was already consolidated, and there was no screw protrusion through the dorsal radial cortex. Surgical exploration was performed. A dorsal longitudinal approach through Lister's tubercle and third compartment with extensor retinaculum exposure was done. Extensor pollicis longus was unscathed. The fourth compartment was explored, with evidence of EDC rupture and EIP laceration (Figure 4). Large bone spurs from the dorsal radius were observed below the tendon plane in relation to Lister's tubercle, generating friction with the extensor apparatus. We confirmed no screws were prominent through the dorsal cortex. Dorsal bone spurs were resected, and tenodesis of EDC to the middle finger tendon was performed. A retinacular flap was designed to protect the tendons from the underlying bone. Volar plate was removed using the previous volar approach, and fracture consolidation was evidenced. The patient underwent physiotherapy rehabilitation, wearing a dynamic elastic band splint. At three-month follow-up, the patient had complete flexion and extension of the fingers, with some slight extensor plus noted as she fully flexed her wrist while her fingers were passively flexed.

## 3. Discussion

Reported volar plating complications reach up to 36% [5, 6]. Tendinous injury secondary to distal radius plating has been strongly related with implant failure (plate positioning, screw length and orientation, and drill violation of dorsal cortex). During a previous study in our institution [7], among 992 consecutive patients treated with volar locking plate, 1.3% developed extensor tendonitis, with no extensor ruptures. Four patients had dorsal tendon irritation related to screw protrusion and 4 patients without dorsal screw protrusion.

According to reports, extensor pollicis longus (EPL) remains the most frequently injured extensor tendon involving distal radius volar plating. Furthermore, EPL rupture without evidence of trauma or pathologic condition has been reported to be caused by a prominent and sharp Lister's tubercle [8]. On the other hand, EDC and EIP injury is much less common [9–11].

According to Wei et al. [12] metaanalysis, the volar approach is more related to neuropathy and carpal tunnel syndrome, while tendon injuries appear to have a stronger relation with the dorsal approach. Azzi et al. [13] showed in their systematic review that tendon rupture involving volar approaches was 1.5%, without differentiating between flexors or extensors. They also report that 0.8% were EPL injuries, while EDC injuries represented only 0.02%. When intrasurgical fluoroscopy or immediate postoperative X-rays reveal some sort of risk factors (screw prominences or misplaced implant) for tendinous injury, early hardware removal is recommended [6].

Surgical techniques for treatment of distal radius fractures dismiss the importance of reduction of metaphyseal dorsal fragments. This condition is indeed a cause of fracture instability when present, but they are usually left in place and have no clinical or imaging implications. We found no reports in medical literature addressing extensor tendon injuries (especially EDC and EIP) as a complication of volar plating for distal radius fracture due to dorsal exostoses. Though we cannot affirm that the cause of tendon rupture in the index case is attrition by dorsal bone spurs, the described complication suggests that hand surgeons should be aware in distal radius fracture with dorsal metaphyseal comminution. Small dorsal fragments maintained by the dorsal periosteum usually have no further sequel if left in place and need no reduction maneuvers. If these fragments protrude dorsally or seem to be rotated perpendicular to the dorsal cortex, the extensor tendons could be in danger and at least attempts for percutaneous reduction could be indicated to avoid future bone spurs formation.
